# Serum Insulin-like Growth Factor-1 Is a Biomarker of Testosterone Production and Intact Acrosome in Asian Elephants (*Elephas maximus*)

**DOI:** 10.3390/ani12121570

**Published:** 2022-06-17

**Authors:** Yuqing Yang, Junpen Suwimonteerabutr, Taweepoke Angkawanish, Kaywalee Chatdarong

**Affiliations:** 1 Research Unit of Obstetrics and Reproduction in Animals, Department of Obstetrics, Gynaecology and Reproduction, Faculty of Veterinary Science, Chulalongkorn University, Bangkok 10330, Thailand; yuqingyang0801@gmail.com (Y.Y.); suwipen@hotmail.com (J.S.); 2 The International Graduate Program of Veterinary Science and Technology, Faculty of Veterinary Science, Chulalongkorn University, Bangkok 10330, Thailand; 3 The Thai Elephant Conservation Center, National Elephant Institute of Thailand, The Forest Industry Organization, Lampang 52190, Thailand; taweepoke@gmail.com

**Keywords:** Asian elephants, biomarker, ELISA, insulin-like growth factor-1, semen parameters, testosterone

## Abstract

**Simple Summary:**

In Thailand, the low fertility rate of Asian elephants has been identified. Factors contributing to poor semen quality in the elephants are not fully understood. Insulin-like growth factor-1 (IGF-1) is related to male infertility. It plays an essential role in testicular development by stimulating cell proliferation, differentiation, and steroidogenesis. In addition, there is increasing evidence that IGF-1 plays a critical role in spermatogenesis. This may be conducive to finding the causes of poor sperm quality in Asian elephants (*Elephas maximus*). In the present study, we investigated the relationships among serum IGF-1, serum testosterone level, and semen parameters in seven elephant bulls. The findings suggest that serum IGF-1 concentration is likely to predict sperm quality like acrosome integrity. The further mechanism by which IGF-1 affects sperm quality requires further investigation.

**Abstract:**

The objective of this study was to find relationships among serum IGF-1, serum testosterone, seminal plasma IGF-1 concentrations and semen parameters in Asian elephants (*Elephas maximus*). A total of 17 ejaculates (one to three ejaculates/bull) were collected from seven captive elephant bulls by performing rectal massage. Before each ejaculation, blood samples were obtained for serum IGF-1 and testosterone assays. Subsequently, the semen characteristics of each ejaculate were evaluated. Mean serum IGF-1 concentration of elephant bulls was estimated as 326.3 ± 114.6 ng/mL (median, 286.2 ng/mL; range, 167.4–542.7 ng/mL). An increase in serum IGF-1 concentration was found to correlate with the percentage of spermatozoa with intact acrosomes. In addition, IGF-1 concentration was positively correlated with testosterone level. However, seminal IGF-1 concentrations could not be detected. In conclusion, our findings suggest that serum IGF-1 concentration is likely a biomarker of normal testicular functions, particularly spermatogenesis in elephants. Moreover, this commercial IGF-1 ELISA is eligible for analyzing serum IGF-1 concentration in Asian elephants.

## 1. Introduction

Captive Asian elephants (*Elephas maximus*) are important not only for the tourism economy but also for improving the genetic diversity of the *ex situ* population. Despite their importance, the number of captive elephants dramatically decreased by 95% in the last century in Thailand [1]. In countries other than Europe, the populations of Asian elephants are not self-sustainable, possibly due to the low birth rates, high mortality and imbalance of sex ratios [2,3]. It has been challenging to obtain optimal sperm quality consistently because high variations in sperm quality have been noted between bulls and even among ejaculates from an individual [4,5,6].

Insulin-like growth factor-1 (IGF-1) plays an essential role in testicular development [7,8]. In the testis, IGF-1 is produced by Sertoli cells and Leydig cells, and it can modulate reproductive performance by stimulating cell proliferation, cell differentiation and steroidogenesis [9,10,11,12,13]. IGF-1 receptors have been identified in the spermatogonia [14], early spermatids [12], spermatocytes [15], spermatozoa [16,17] and cells of epididymis [18,19], indicating that IGF-1 may be involved in steroidogenesis and signals regulating spermatogenesis possibly via the paracrine-autocrine system. Recently, the serum IGF-1 concentration in association with sperm concentration and motility has been identified in buffaloes [20]. During puberty in beef bulls, IGF-1 level in peripheral blood is used as an indicator to predict aberrant sperm motility [21]. In men, the serum IGF-1 level was associated with normal sperm morphology and percentage of sperm motility [22]. It has been shown that the addition of IGF-1 into seminal plasma can improve sperm quality [21,23,24,25]. Furthermore, in the seminal plasma, IGF-1 has been investigated in boars [23,26], cattle [16], stallions [15] and humans [17,22]. Seminal IGF-1 concentration appears to be related to greater semen quality [27,28].

Semen collection from elephants requires an experienced team to handle the complex procedures. In addition, elephants need to be trained well. Therefore, measuring a serum biomarker for semen quality would be a helpful step prior to selecting a potential semen donor. The objectives of this study were to: (i) determine the relationships among serum IGF-1, serum testosterone level and semen parameters, and (ii) assess the validity of a commercial human sandwich IGF-1 enzyme-linked immunosorbent (ELISA) kit for detecting concentrations of serum IGF-1 in this endangered species.

## 2. Materials and Methods

### 2.1. Animals

The experimental protocol was ethically approved by the Animal Care and Use Committee, Faculty of Veterinary Science, Chulalongkorn University, Bangkok (approval number 2031044).

Seven healthy captive-born Asian elephant bulls (E1, E2, E3, E4, E5, E6 and E7), aged from 12 to 47 years (averaged 29.1 ± 13.8 years) with a mean bodyweight of 3357 ± 512 kg, housed at the National Elephant Institute (NEI), Forest Industry Organization Lampang, Thailand (latitude 18°21.60′ E and longitude 99°14.92′ E) were included. All samples were obtained in June 2020 to July 2020. All animals were maintained in mixed social groups with females. They worked for tourists either riding (with a saddle) or showing for no more than 4 h per day. When not working, the elephants were fed with Napier grass (*Pennisetum purpureum*), Pangola grass (*Digitaria eriantha*), tamarind (*Tamarindus indica*), banana and other natural foods. The availability of foods varied among seasons. After working, all bulls were chained separately with long 20–30 m chains in the forest for foraging (from 16:00 to 18:30). Routine health examination was performed by veterinarians three times a year.

### 2.2. Blood Collection, Semen Collection and Processing Procedures

Blood samples (3–6 mL; *n* = 17) were collected from elephant ear veins between 9:00 and 10:00 a.m. before each ejaculation. Blood was allowed to coagulate at room temperature (RT) for 1–2 h and centrifuged at 2000× *g* for 10 min. Serums were harvested and stored at −20 °C until analyzed in a single batch.

Seventeen ejaculates (one to three ejaculates per bull) were obtained using the rectal massage method as previously described [29]. Briefly, each bull was restrained in a chute. After removing the feces, collecting containers were placed over the penis. Then, the pelvic portion of the urethra and ampulla were massaged.

### 2.3. Semen Evaluation

Ejaculates with visualized or olfactory scents of urine contamination were excluded. Each ejaculate was evaluated immediately for color, volume and pH. For evaluations of motility and progressive motility, 5 µL of semen was dropped onto a pre-warmed glass slide (37 °C) and covered with a pre-warmed coverslip. Percentages of motility and progressive motility were subjectively assessed under a phase-contrast microscope at a magnification of 200× (Olympus, Shinjuku, Japan) by 3 independent evaluators. A hemocytometer chamber (Boeco, Hamburg, Germany) was used to determine sperm concentration.

#### 2.3.1. Sperm Membrane Integrity Assessment

Eosin-nigrosin staining was used to assess the percentage of viable sperm. A total of 200 spermatozoa were evaluated. Spermatozoa with no staining were classified as an intact membrane (or live spermatozoa) and spermatozoa-stained pink were classified as a non-intact membrane (or dead sperm) [30].

#### 2.3.2. Sperm Functional Membrane Integrity Assessment

The functional integrity of the sperm plasma membrane was determined using a short hypo-osmotic swelling test (sHOST) [31,32]. Briefly, each ejaculate (20 µL) was incubated at 37 °C in 200 µL hypo-osmotic solution (75 mOsm/kg; 1:10, *v*/*v*) for 1 h. After incubation, the semen was fixed in a hypoosmotic solution supplemented with 5% (*v*/*v*) formaldehyde (Merck, Darmstadt, German). A drop (5 µL) of the mixture was placed on a warm slide (37 °C) and covered with a coverslip. A total of 200 spermatozoa per sample were counted under a phase-contrast microscope (400×). Spermatozoa presenting coiled tails were classified as intact functional membrane integrity (sHOST positive).

#### 2.3.3. Sperm DNA Integrity Assessment

Sperm DNA integrity was examined on Acridine orange (AO) fluorescence by modifying previous reports [33,34]. Briefly, 8–10 µL of diluted semen was smeared onto a glass slide and air-dried on a slide warmer (37 °C). The slides were fixed in Carnoy’s solution (methanol:glacial acetic acid, 3:1 *v*/*v*) overnight at RT. The smeared slides were air-dried and stained with 1% (100 mg/mL) AO for 10 min. The AO staining solution was prepared daily by adding 10 mL of 1% AO to 40 mL of 0.1 M citric acid (Merck, Darmstadt, Germany) and 2.5 mL of 0.3 M Na_2_HPO_4_·7H_2_O (Merck, Darmstadt, Germany) and then stored at RT in the dark. After staining, the slides were gently rinsed in a stream of distilled water. In total, 200 spermatozoa per slide were examined using a fluorescent microscope (BX51, Olympus, Tokyo, Japan) at a 1000× magnification with oil immersion. Spermatozoa heads with green fluorescence were considered as DNA intact (double-stranded), whereas those spermatozoa stained with the red, yellow, orange or mixed fluorescence were considered a DNA damaged.

#### 2.3.4. Acrosome Integrity Assessment

The intactness of sperm acrosome was evaluated using fluorescein isothiocyanate-labeled peanut (*Arachis hypogaea*) agglutinin (FITC-PNA) combined with propidium iodide (PI) staining [35]. Briefly, 10 µL FITC-PNA (1 mg/mL) was diluted with 90 µL phosphate buffer saline (PBS) and mixed with 5 µL of 340 µM PI. A circle smear of 5 µL of diluted semen was performed on a clean slide and air-dried. The sample was fixed in 95% ethanol (*v*/*v*) for 30 s and allowed to air dry. Subsequently, a 50 µL mixture of FITC-PNA/PI was spread over the smeared slide. After incubation for 30 min in a dark humidified chamber at 4 °C, slides were rinsed with cold distilled water and air-dried. A total of 200 spermatozoa per sample were examined under a fluorescent microscope at a 1000× magnification with immersion oil. Only acrosome-intact sperm were stained with a bright green fluorescence [35,36].

### 2.4. Validation and Evaluation of Serum IGF-1

Serum IGF-1 concentrations were determined by a commercial sandwich enzyme-linked immunosorbent assay (Human IGF-1 ELISA E20, Mediagnost, Germany) which was first used on the elephants. The genome of the African elephant (*Loxodonta africana*) was searched by the Broad Institute (http://www.broadinstitute.org). A Blast search (accessed on 22 August 2020) from NCBI revealed human and elephant predicted IGF-1 homology of the amino acid sequence was 98% matched to humans, except for number 67 [37].

Before running the assay, serum samples were diluted with sample buffer at a 1:21 dilution ratio according to the manufacturer’s protocol. Briefly, 80 µL of goat biotinylated anti-IGF-1 antibody was added to each well first. Subsequently, 20 µL of blank, controls, standards and diluted samples were added to the wells of the microtiter. The plate was incubated for 1 h at RT and washed firstly with washing buffer 5 times. After washing, horseradish peroxidase (HRP)-labeled streptavidin was added to each well. After 30 min incubation and washing again, tetramethylbenzidine (TMB)-substrate was added and incubated for 15 min. The reaction was terminated by adding sulphuric acid solution. The absorbance was measured at 450 nm immediately using an ELISA plate reader (Tecan Sunrise^TM^, Männedorf, Switzerland).

To measure precision in the ELISA, intra-assay, inter-assay coefficient of variation (CVs), and total imprecision assay CV of 3 elephant serum samples having low, medium and high concentrations were calculated. The inter-assay CV was calculated by analyzing the same samples in duplicate on 4 separate ELISA plates on 4 different days. The intra-assay CV was evaluated from the CV of the same samples three times in duplicates. The total imprecision assay CV(CV_total_) was calculated: CV_total_ $=\sqrt{\mathrm{CV}{\left(\mathrm{inter}\right)}^{2}+\mathrm{CV}{\left(\mathrm{intra}\right)}^{2}}$ [38]. Acceptable intra- assay and inter-assay CV were less than 10% and 15%, respectively. Desirable and acceptable CV_totals_ were set at 8.5% and 12.75%, respectively [38].

Linearity-of-dilution assay and spike-and-recovery tests were designated simultaneously for representing accuracy indirectly. A serum sample was chosen for demonstrating the concentration of IGF-1 (254.6 ng/mL). The linearity under dilution was evaluated by diluting (1:2 to 1:8) this serum sample serially with PBS. Expected concentration values were calculated by dividing the undiluted sample by the dilution factor [38,39]. The percentage of recovery is calculated by comparing observed and expected values. In the spike-recovery test, five low-concentration serum IGF-1 samples were pooled [40]. The pooled sample was then mixed with 100 µL of 2, 5, 15 and 30 ng/mL of standard recombinant human IGF-1 in duplicates. Recovery of spiked IGF-1 was calculated according to the previous studies: [spiked sample (ng/mL)/(neat (ng/mL) + spike solution (ng/mL)] * 100 with an acceptable recovery range from 80% to 120% [38].

### 2.5. Evaluation of Serum Testosterone

Testosterone in serum was analyzed using an enzyme immunoassay (EIA) at the Endocrine Laboratory, Conservation Research and Animals Health, Khao Kheow Open Zoo, Chon Buri, Thailand. This EIA employs a polyclonal antiserum (R156/7; supplied by C. Munro, University of California, Davis, CA, USA) and HRP conjugated testosterone label (C. Munro) by following the previous studies [40,41]. Briefly, 96-well plates were pre-coated with antiserum (50 µL; 1:8500 dilution) in coating buffer (0.05 M NaHCO_3_, pH 9.6). Standards (50 µL; values, 600, 300, 150, 75, 37.5, 18.75, 9.37, 4.68 and 2.34 pg/well) diluted in dilution buffer (0.1 M NaPO_4_, 0.149 M NaCl, 0.1% bovine serum albumin (BSA), pH 7.0) and serum samples (50 µL) were mixed with HRP (50µL; 1:80,000 dilution). After incubation at RT for 2 h, plates were washed four times with wash buffer. Then, 100 µL of TMB substrate solution was added to each well and incubated at RT for 45–60 min. After adding the stop solution, the plate was read immediately at 450 nm on a plate reader (Tecan^®^ Infinite F50, Männedorf, Switzerland). Inter- and intra-assay CV were less than 10%, respectively. The testosterone sensitivity was 46 pg/mL.

### 2.6. Statistical Analysis

Data were expressed as mean ± SD. Statistical analyses were performed using SPSS (IBM SPSS statistics version 26.0.0.0, SPSS Inc., Chicago, IL, USA). The normality of distribution was measured by the Kolmogorov Smirnov test and confirmed by Qualitative-Quantitative plots (Q-Q plots). The levels of serum testosterone and IGF-1 with semen analysis results were presented using descriptive statistics. Pearson’s correlation coefficient (*r*) was applied to find correlations among serum IGF-1, serum testosterone level and all parameters. The significance was set at *p* < 0.05 with a confidence interval of 95%.

## 3. Results

The background information including the mean values of serum IGF-1 and testosterone of individual Asian elephant bulls is summarized in Table 1. In the present study, IGF-1 concentrations (range, 167.4–542.7 ng/mL; mean, 326.3 ± 114.6 ng/mL) were highly varied among bulls. The youngest elephant E1 (12 years old) in the group had a higher serum IGF-1 concentration (average at 473.6 ng/mL) while the oldest E4 had the lowest level (average at 215.0 ng/mL). The mean (±SD) concentration of serum testosterone was 1190.1 ± 1081.8 pg/mL (range, 53.2–3150.2 pg/mL). The testosterone of the E4 was not detected. In the current study, the same protocol and ELISA kit were applied to analyze seminal plasma IGF-1 of Asian elephants. No seminal IGF-1 concentrations were successfully detected. We attempted to create a new standard curve by following the previous study [16]. However, IGF-1 concentrations were still not determined.

The mean values of sperm parameters and correlations among serum IGF-1, testosterone concentration and semen parameters in this study are shown in Table 2. There was a positive correlation between serum IGF-1 and testosterone concentration (*r* = 0.73, *p* = 0.004, Table 2). Additionally, positive correlations between acrosome integrity (*r* = 0.53, *p* = 0.028) and IGF-1 concentration were demonstrated.

**Figure 1:**
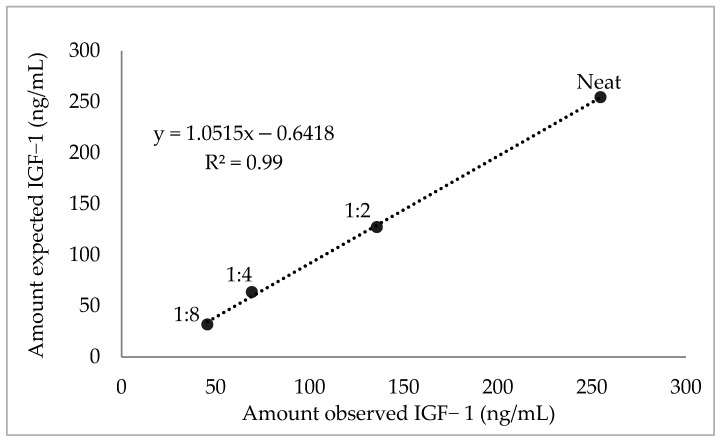
Investigated linearity through a serial dilution of one serum sample to demonstrate an accurate linear detection of examined insulin-like growth factor-1 (IGF-1) concentrations. R^2^ value represents the coefficient of determination.

The linearity under the serial dilutions was detected (R^2^ = 0.99; Figure 1) and the recovery rate ranged from 100 to 143% with a mean of 114.7 ± 19%, which was acceptable. Inter-assay, intra-assay and total imprecision CV for low, medium and high concentration samples were 1.6–6.4%, 4.7–6.9% and 7.1–8.0%, respectively. In spike-recovery assays, the overall mean recovery of spiked IGF-1 was acceptable (mean 107.2 ± 21%, range 80.8–136.9%).

## 4. Discussion

In the current study, the relationships between serum IGF-1, serum testosterone concentration and intact acrosome of spermatozoa were demonstrated in Asian elephants. Corresponding to the fact that IGF-1 is produced by Sertoli and Leydig cells in the testis, the correlations with testosterone level and acrosome integrity in this study indicated the role of IGF-1 in supporting spermatogenesis. Serum IGF-1 of the male white-tailed deer peaks during the breeding season, suggesting it probably plays a critical role in the reproduction [43]. Because assessment of initial sperm motility cannot be a reliable sole indicator of semen quality [43,44], the serum IGF-1 level may be beneficial as an additional biomarker to assess breeding soundness in elephants. The IGF-1 values were found to vary among species; the value for buffalo bulls (mean, 1555.22 ng/mL; range, 927–2247 ng/mL) evaluated using an ELISA was much higher than that of deer (63.6 ng/mL; 5.81–224.9 ng/mL) [45] and men (mean, 175.4 ± 42.7 ng/mL) [22] both of which were assessed using radioimmunoassay (RIA) as well as male Asian elephants (326.3 ng/mL; 167.4–542.7 ng/mL) in the current study.

The present results revealed that serum IGF-1 concentration was positively correlated with the proportion of spermatozoa with intact acrosomes in this endangered species. Abnormality of acrosomal morphology has been implicated in threatening fertility rate in the boars during artificial insemination procedures [46] and in humans during the *in vitro* fertilization (IVF) process [47]. Previous studies have validated that the IGF-1 receptor is mainly localized in the acrosomal region of the bovine and human sperm [12,17], suggesting that IGF-1 may be required in this region for capacitation and the acrosome reaction processes. In addition, IGF-1 purified from seminal plasma can induce the acrosome reaction in capacitated spermatozoa of rabbits [48], also suggesting that IGF-1 plays a key role in sperm capacitation. In the buffaloes, supplementation of IGF-1 in the semen provides a positive effect on maintaining the integrity of the sperm acrosomal membrane [49]. However, the precise mechanism in which the way IGF-1 directly affects sperm quality is not clearly understood.

Similar to the reports in the deer [45] and cattle [21], serum IGF-1 in the present study was found to correlate positively with serum testosterone levels. In men, serum IGF-1 is simultaneously increased with the testicular size [50]. IGF-1 [15,51] and testosterone [52] are produced in the testis and appear to regulate sperm head formation during spermatogenesis via endocrine, paracrine and autocrine systems [53,54]. In Leydig cells, testosterone stimulates IGF-1 production and IGF-1 binding proteins (IGFBPs) [55], which in turn regulates the normal function of Sertoli cells [13,56]. Mice with an Igf1 null mutation gene are observed to have smaller testis and epididymis sizes, and lower levels of testosterone and sperm concentration compared to the controls [57]. Similar findings demonstrated in deer that circulating serum IGF-1 concentration is associated with serum testosterone level [45]. The above findings, which are similar to our results, demonstrate the potential relationships between IGF-1 and testosterone in the testis.

The variations in serum IGF-1 concentrations among elephant bulls were likely affected by the different diets [58,59,60]. After work, the elephants in this study were allowed to forage in different areas where the availability (quantity and quality) of food was varied across the areas. However, all the bulls were classified as having normal body condition scores (BCS = 3.0–3.5). In addition to the diets, age has been suggested to contribute to the IGF-1 variations in other species; specifically, concentrations of blood IGF-1 peak during puberty and decrease after puberty and reach a low level around old age [61,62]. Captive male elephants reach puberty at 10–15 years old [63] with the highest serum testosterone [5]. As a consequence, it is not surprising that the elephant E1 (12 years old) had the highest level of serum IGF-1 and testosterone whereas E4 (47 years old) had the lowest IGF-1 in the present study. Furthermore, it has been previously reported that serum testosterone is the lowest when Asian elephants are old in Thailand [5], which could explain why the testosterone concentration of this elderly E4 was not detected.

To the best of our knowledge, this is the first report of using validating a commercial ELISA to successfully measure the serum IGF-1 in Asian elephants. The mean value of elephant bull serum IGF-1 (326.3 ng/mL) in the present study was similar to the previous report (307 ng/mL) detected using an RIA which is not acceptable since radioactive substances are used [64]. In the validation process of serum IGF-1, the acceptable R^2^ value and the recovery rate of serial dilutions were acceptable, suggesting a high accuracy of the linearity test. The inter- (<10%), intra-assay (<10%) and total imprecision CV (<8.5%) indicated high precision. However, this study had limitations. The sample size was likely small because elephant restraint and semen collection require huge resources including manpower. Most of the IGF-1 concentrations in the seminal plasma analyzed using the same ELISA kit were below the minimum detection range of the ELISA kit. An extraction process to concentrate the seminal IGF-1 might be helpful. Additionally, it is necessary to analyze IGF-1 concentration as soon as possible because IGF-1 in blood degrades dramatically during storage at both −20 and −80 °C for one month, not to mention three months [65].

## 5. Conclusions

Serum IGF-1 concentration is likely a reliable biomarker for acrosome integrity. The evidence of serum testosterone concentrations confirmed the role of IGF-1 in supporting spermatogenesis in Asian elephant bulls.

## Figures and Tables

**Table 1:** Summary of age, body condition score (BCS), bodyweight, total ejaculate number, mean serum IGF-1, testosterone concentration and fertility history of seven Asian elephant bulls.

ID	Age (y)	BCS	Body Weight (kg)	Ejaculate Number	Mean IGF-1 (ng/mL)	Mean Testosterone (pg/mL)	Fertility History
E1	12	3	2690	2	437.6	1645.0	No
E2	20	3.5	3490	4	300.4	138.3	No
E3	27	3.5	4055	2	271.4	1914.6	No
E4	47	3	3460	4	215.0	Not detected	No
E5	27	3	3000	2	283.9	576.5	No
E6	46	3	3210	2	541.6	2996.8	Yes
E7	40	3	4070	1	323.7	652.77	No

Body condition score (BCS): 5-point scale (1–5, thinnest–fattest); 3–3.5 was defined as normal BCS [42].

**Table 2:** Correlations among semen parameters, serum testosterone and IGF-1 concentration with mean values (±*SD*) of seven elephant bulls.

Parameters	Mean	Serum IGF-1 (*r*)	*p* Value	Serum Testosterone (*r*)	*p* Value
Semen volume (mL)	32.1 ± 13.3	0.12	0.66	0.16	0.60
Semen pH	7.3 ± 0.7	0.27	0.29	0.05	0.87
Sperm concentration (×10^6^/mL)	1209.2 ± 403.4	0.07	0.80	0.24	0.41
% Motility	32.6 ± 22.8	0.09	0.74	0.18	0.55
Progressive motility (1–5)	3.2 ± 1.4	−0.15	0.58	0.03	0.92
% Membrane integrity	58.6 ± 21.0	0.07	0.80	0.16	0.61
% Intact functional membrane	26.5 ± 16.8	0.09	0.74	0.11	0.73
% Normal DNA integrity	70.0 ± 23.5	0.13	0.61	0.02	0.94
% Intact acrosome	44.5 ± 18.3	0.53	0.028 *	0.22	0.47
Serum testosterone (pg/mL)	1190.1 ± 1081.8	0.73	0.004 **	-	-
Serum IGF-1 (ng/mL)	32 6.3 ± 114.6	-	-	0.73	0.004 **

Abbreviations: % Motility = the percentage of sperm motility; % Membrane integrity = the percentage of sperm membrane integrity; % Intact functional membrane = the percentage of sperm functional membrane integrity; % Normal DNA integrity = the percentage of normal sperm DNA; % Intact acrosome = the percentage of acrosomal integrity; IGF-1 = insulin-like growth factor-1. * means *p* < 0.05; ** means *p* < 0.01. Semen samples: *n* = 17 ejaculates; Serum IGF-1 samples: *n* = 17; Serum testosterone samples: *n* = 17.

## Data Availability

Not applicable.
